# Comprehensive Transgender Healthcare: The Gender Affirming Clinical and Public Health Model of Fenway Health

**DOI:** 10.1007/s11524-015-9947-2

**Published:** 2015-03-17

**Authors:** Sari L. Reisner, Judith Bradford, Ruben Hopwood, Alex Gonzalez, Harvey Makadon, David Todisco, Timothy Cavanaugh, Rodney VanDerwarker, Chris Grasso, Shayne Zaslow, Stephen L. Boswell, Kenneth Mayer

**Affiliations:** 1 The Fenway Institute, Fenway Health, 1340 Boylston Street, 8th Floor, Boston, MA 02215 USA; 2 Department of Epidemiology, Harvard School of Public Health, Boston, MA USA; 3 Department of Community Health Sciences, Boston University School of Public Health, Boston, MA USA; 4 Transgender Health Program, Medical and Behavioral Health Departments, Fenway Health, Boston, MA USA; 5 The Danielsen Institute, Boston University, Boston, MA USA; 6 Department of Medicine, Beth Israel Deaconess Medical Center and Harvard Medical School, Boston, MA USA; 7 Executive Office, Fenway Health, Boston, MA USA; 8 Department of Global Health and Population, Harvard School of Public Health, Boston, MA USA

**Keywords:** Health equity, Health care, Transgender

## Abstract

This report describes the evolution of a Boston community health center’s multidisciplinary model of transgender healthcare, research, education, and dissemination of best practices. This process began with the development of a community-based approach to care that has been refined over almost 20 years where transgender patients have received tailored services through the Transgender Health Program. The program began as a response to unmet clinical needs and has grown through recognition that our local culturally responsive approach that links clinical care with biobehavioral and health services research, education, training, and advocacy promotes social justice and health equity for transgender people. Fenway Health’s holistic public health efforts recognize the key role of gender affirmation in the care and well-being of transgender people worldwide.

## Introduction

Transgender people have an assigned sex at birth that differs from their current gender identity or expression.^1^ This report describes the evolution of Fenway Health’s multidisciplinary model of transgender health care, research, education, training, and dissemination of its practice. This includes the development of, and changes to, a community-based approach spanning almost two decades. Opportunities for future growth of transgender care and research locally and globally are discussed, with a focus on the linkage of clinical care with health research, education, training, and advocacy to promote social justice and health equity for transgender people across the world.

## About Fenway Health

The mission of Fenway Health is to enhance the well-being of the lesbian, gay, bisexual, and transgender (LGBT) community and all people in local neighborhoods and beyond.^2^ Fenway Health was founded as a grassroots clinic in 1971 in a central urban neighborhood of Boston, Massachusetts, as part of a grassroots effort in the heyday of political activism. Since that time, Fenway Health has developed into a comprehensive federally qualified community health center offering integrated primary medical and behavioral health services with an emphasis on lesbian, gay, bisexual, and transgender (LGBT) health. In 1975, the center recorded 5000 patient care visits.^3^ In 2013, Fenway Health providers saw 21,600 patients who made 110,000 patient visits.^2^ In the early 1980s, Fenway’s response to the AIDS epidemic included the development of the first community-based HIV research program in New England, which ultimately led to the development of The Fenway Institute, an embedded community-based research entity, colocated in a community health center. Fenway Health’s annual budget exceeds $65 million with more than 600 staff people employed there. Fenway Health is the largest care provider and employer of transgender people in Massachusetts.

## Transgender Health Program

### History and Initial Growth

**FIG. 1:**
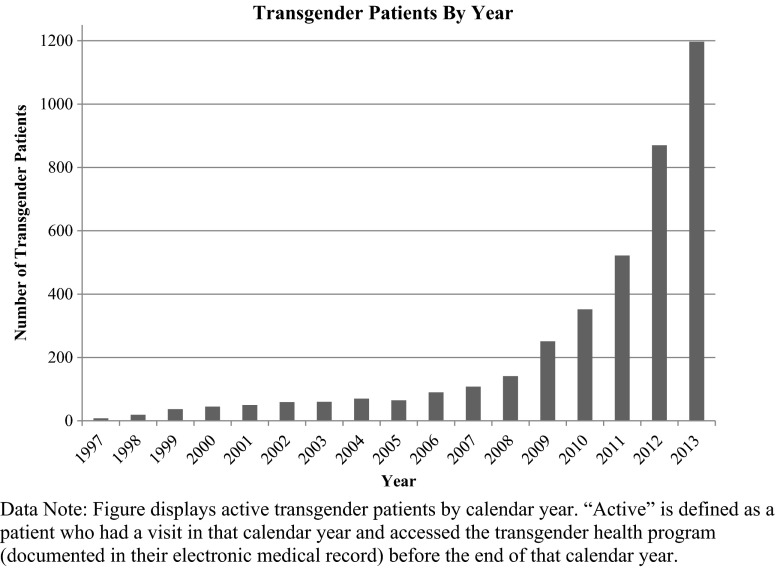
Annual growth of transgender patient population at Fenway Health from 1997 to 2013. Data note: figure displays active transgender patients by calendar year. “Active” is defined as a patient who had a visit in that calendar year and accessed the transgender health program (documented in their electronic medical record) before the end of that calendar year.

Transgender people have sought health care and been served by Fenway Health since the organization’s initial founding. As Fenway’s patient base grew in the 1980’s and early 1990’s, the organization recognized gaps in non-HIV healthcare services. One of these gaps was access to medical gender affirmation services (e.g., cross-sex hormone therapy), and the provision of other optimal, culturally tailored services for transgender people. Fenway’s Board of Directors and leadership recognized the growing need for comprehensive care for transgender people and decided that this was a priority area for the organization. “T” in “LGBT” was officially added to Fenway’s organizational mission in 1995. Hiring of staff specifically trained to focus on the needs of this community led to the development of the Transgender Health Program. Fenway’s transgender patient population steadily increased in the first decade from 8 patients in 1997 to 90 patients in 2006 and more than 1200 by 2014 (Fig. 1).

Fenway’s organizational systems expanded during this time to support providers and other staff in delivering gender affirmative care. For example, routine data capture in electronic health record fields was implemented to gather preferred name, pronouns, assigned sex at birth, and current gender identity of patients, data important for providing gender affirmative care in addition to assessing the quality of care provided to transgender patients. Cultural competency trainings related to transgender health were implemented throughout the organization, including modules that became part of new staff orientation and regularly part of ongoing departmental trainings.

### Pioneering a Modified Informed Consent Model of Transgender Care

In 2007, Fenway implemented a modified informed consent model for cross-sex hormone therapy pioneering the dissemination of an accessible, holistic, gender affirming, and multidisciplinary model of transgender care. The informed consent model removed unnecessary barriers to hormone therapy, including restrictions specifying prolonged mental health evaluations and “real life tests” (i.e., living full-time in one’s self-identified gender) to obtain hormone therapy that had long been embedded in existing standards of care. Transgender patients were asked to complete a hormone readiness assessment before accessing gender affirmation services, but mental health counseling was not automatically required. During this timeframe, Fenway Health also relocated to a full-service 10-story facility dedicated to LGBT health, increasing its capacity to serve more transgender patients.

With the implementation of a modified informed consent model, Fenway’s transgender patient population grew exponentially (Fig. 1). The menu of services provided to transgender patients at Fenway Health is outlined in Table 1. Demographic characteristics of active transgender patients served at Fenway Health in calendar year 2013 are in Table 2, delineated by gender identity (52 % female-to-male and 48 % male-to-female spectrum).

**TABLE 1:** Services provided as part of Transgender Healthcare at Fenway Health

• Primary care for adults and adolescents: Internal Medicine, Family Medicine
• Family planning: alternative insemination, contraception counseling and administration
• Transgender care: reproductive rights and medication counseling, hormone administration and monitoring, post-operative wound/drain/prosthesis care
• HIV and STD care: comprehensive HIV/AIDS care and case management, HIV/STD counseling and testing, STD treatment and partner notification services
• Internal Medicine subspecialty care: pulmonology, infectious disease
• Other medical specialties: obstetrics, gynecology, podiatry, nutrition
• Complementary and alternative medicine specialties: acupuncture, massage therapy, osteopathic manipulative therapy
• Integrated behavioral health and non-clinical case management
• Diagnostic imaging: digital x-ray and mammography, bone mineral density screening
• Clinical and retail pharmacy
• General dentistry
• Optometry

**TABLE 2:** Demographic characteristics of the transgender patient population at Fenway Health served in calendar year 2013 (*n* = 1197)

TOTAL	Female-to-male (FTM)		Male-to-female (MTF)		Total	
	*N*	%	*N*	%	*N*	%
	621	*52* %	576	*48* %	1197	*100* %
Age	FTM		MTF		Total	
Mean age (in years)	28.52		35.15		31.71	
Gender identity	FTM-N	FTM-%	MTF-N	MTF-%	Total-N	Total-%
Female	8	1 %	244	42 %	252	21 %
Male	283	46 %	29	5 %	312	26 %
Genderqueer or not exclusively male or female	87	14 %	47	8 %	134	11 %
Missing/not reported	243	39 %	256	44 %	499	42 %
Race/ethnicity	FTM-N	FTM-%	MTF-N	MTF-%	Total-N	Total-%
White non-Hispanic	494	80 %	384	67 %	878	73 %
Black	35	6 %	49	9 %	84	7 %
Latino/Hispanic	29	5 %	60	10 %	89	7 %
Asian	24	4 %	31	5 %	55	5 %
Multiracial	25	4 %	26	5 %	51	4 %
Other	10	2 %	16	3 %	26	2 %
Missing/not reported	4	1 %	10	2 %	14	1 %
Employment status	FTM-N	FTM-%	MTF-N	MTF-%	Total-N	Total-%
Employed: unspecified	102	16 %	88	15 %	190	16 %
Employed: part-time	28	5 %	12	2 %	40	3 %
Employed: full-time	36	6 %	17	3 %	53	4 %
Employed: self-employed	1	0 %	2	0 %	3	0 %
Unemployed	18	4 %	22	4 %	40	3 %
Retired	1	0 %	2	0 %	3	0 %
Student	56	9 %	38	7 %	94	8 %
Other	7	1 %	10	2 %	17	1 %
Not reported	372	60 %	385	67 %	757	63 %
Income category	FTM-N	FTM-%	MTF-N	MTF-%	Total-N	Total-%
100 % of poverty level and below	277	45 %	245	43 %	522	44 %
101–150 % of poverty level	33	5 %	31	5 %	64	5 %
151–200 % of poverty level	26	4 %	19	3 %	45	4 %
Over 200 % of poverty level	115	19 %	89	15 %	204	17 %
Refused to report	56	9 %	69	12 %	125	10 %
Missing/unknown	114	18 %	123	21 %	237	20 %
Insurance	FTM-N	FTM-%	MTF-N	MTF-%	Total-N	Total-%
Medicaid	146	24 %	200	35 %	346	29 %
Medicare	36	6 %	88	15 %	124	10 %
Missing	6	1 %	4	1 %	10	1 %
None/uninsured	27	4 %	37	6 %	64	5 %
Private	406	65 %	247	43 %	653	55 %
Hormones	FTM-N	FTM-%	MTF-N	MTF-%	Total-N	Total-%
Yes	503	81 %	513	89 %	1016	85 %
No	118	19 %	63	11 %	181	15 %
Surgery	FTM-N	FTM-%	MTF-N	MTF-%	Total-N	Total-%
Yes	254	41 %	123	21 %	377	31 %
No	128	21 %	102	18 %	230	19 %
Unknown/not reported	239	38 %	351	61 %	590	49 %
Months at Fenway	FTM		MTF		Total	
Mean length being a patient (in months, since first visit)	35.87 months (3 years)		42.64 months (3.5 years)		39.13 months (3 years, 1 month)	

The number of transgender people getting their health care at Fenway continues to grow, and Fenway’s model of care continues to dynamically evolve to meet the needs of the patient population. Underlying this approach is a philosophy of accessible, patient-centered care that views gender affirmation as routine part of primary care service delivery, not a psychological or psychiatric condition in need of treatment. Feedback and input from the transgender community through consultations with key opinion leaders and community forums is used to direct development of service delivery programs to ensure ongoing responsiveness of care. Part of the responsiveness to care needs voiced by the transgender community includes over 80 in-person clinical care and competency trainings of mental health and medical providers regionally and nationally conducted by the Transgender Health Program since 2007.

## The Fenway Institute: Transgender Health Research, Education, Training, and Advocacy

Fenway Health initiated one of the nation’s first community-based HIV research programs in 1983.^2^ Since then, The Fenway Institute (TFI) at Fenway Health has expanded in scope and broadened its mission—to make life healthier for those who are LGBT, people living with HIV/AIDS, and the larger community through research and evaluation, education and training, and public health advocacy, resulting in many multidisciplinary publications. TFI faculty members have been at the forefront of scientific and advocacy efforts to increase knowledge about transgender health.^4^–^7^

Transgender research projects continue to steadily increase and represent a diverse portfolio.^8^–^14^ One area of focus in transgender health research at TFI is on methodological innovations, including data collection and measures for clinical care^15^ and population studies^7^,^16^–^18^ and sampling research^19^ and study designs.^20^,^21^ TFI faculty have researched best methods to gather information in electronic medical records for the care of gender minorities and have advocated nationally for the systematic collection of gender-inclusive data such as preferred name and pronoun, assigned sex at birth, and gender identity.^15^,^22^–^24^

TFI researchers have expanded transgender health collaborations globally, including but not limited to partnerships in Latin America, the Caribbean, Portugal, and Spain, India, Vietnam, and Amsterdam. Funding streams supporting transgender health research are diverse and include the National Institutes of Health (NIH), the Patient-Centered Outcomes Research Institute (PCORI), and amFAR The Foundation for AIDS Research. For example, through NIH funding, TFI researchers are testing a model of asset-based intervention to provide young transgender women ages 16–29 years with skills that will enhance their resiliency and decrease their risk of acquiring or transmitting HIV.^25^ The model has been expanded to culturally tailor the intervention for young transgender men who have sex with men.

**FIG. 2:**
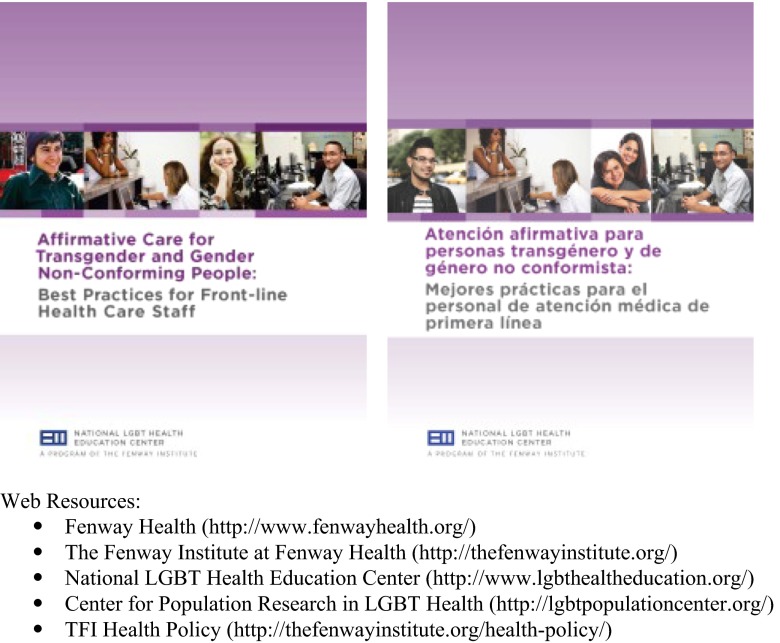
Collaboration between The National LGBT Health Education Center, faculty at The Fenway Institute (TFI), and Transgender Health Program to address cultural competency of front-line health care staff to provide gender affirming care to transgender patients. Web resources: Fenway Health (http://www.fenwayhealth.org/). The Fenway Institute at Fenway Health (http://thefenwayinstitute.org/). National LGBT Health Education Center (http://www.lgbthealtheducation.org/). Center for Population Research in LGBT Health (http://lgbtpopulationcenter.org/). TFI Health Policy (http://thefenwayinstitute.org/health-policy/).

In 2011, the National LGBT Health Education Center at TFI through funding from HRSA began expanded education and training efforts to improve care for transgender patients throughout the country. This included collaboration between TFI and the Fenway’s clinical Transgender Health Program to develop materials focused on improving clinical care center’s front-line staff competency (Fig. 2). The Education Center regularly provides trainings on transgender health to health center-based and hospital-based programs around the nation and has six archived online webinars focused on transgender health needs in diverse populations ranging from the needs of youth to the needs of farmworkers. Collaborations between transgender health researchers nationally and globally are ongoing through the Transgender Working Group at the Center for Population Research in LGBT Health at TFI.

## Conclusion

The rapid and sustained growth of Fenway Health’s transgender health care, research, education, training, and advocacy might be succinctly summarized by the mantra from the movie *Field of Dreams*: “If you build it, they will come.” Clearly there are many who have not had access to care simply because it did not exist. Fenway Health’s model of transgender clinical care has continued to grow exponentially, change, and dynamically evolve to meet the changing needs of local transgender patients and the changing health care delivery system. The development of the most recent World Professional Association for Transgender Health (WPATH) *Standards of Care*, *Version 7* in 2011 was informed in part by the success of Fenway Health’s and other community health centers’ (e.g., Callen Lorde and Tom Waddell) informed consent models of clinical care.^26^,^27^ Fenway Health, alongside several other community health centers, shaped standards for transgender people worldwide. The integration of community assessment, research, education, training, and advocacy alongside clinical care provides a holistic public health model that highlights the key role of gender affirmation in caring for transgender people.

In many ways, the evolution of transgender health at Fenway Health mirrors larger societal changes in conceptualizations of transgender people—shifting away from pathology (transgender as “disorder”) to a strengths-based depathologization of human gender diversity (transgender as “identity”).^28^ Adaptation and replication of Fenway Health’s patient-centered, gender affirming model of transgender clinical care, research, education, training, and advocacy in other settings and contexts represents a unique future opportunity to meet the underserved needs of transgender people. Reducing transgender health inequities globally necessitates foregrounding gender affirmation in health care service delivery. Clearly, many challenges exist to mainstreaming the care of transgender people. Not the least of these are developing treatment algorithms which provide smart reminders to clinicians about the needs of transgender patients, much like electronic systems remind providers to care for basic needs such as monitoring screening with tests like Pap smears and mammography and prostate exams. There is also work to be done with payers to advocate for coverage for transgender-related affirmative care including medical, behavioral, and surgical services in addition to providing for basic primary care needs in an inclusive manner.
